# Pancoast tumours: clinical assessment and long-term results of combined radiosurgical treatment

**DOI:** 10.1186/1749-8090-8-S1-P132

**Published:** 2013-09-11

**Authors:** F Gradica

**Affiliations:** 1 Thorax Surgey Department, University Hospital "Shefqet Ndroqi" Tirana, Albania

## Background

Pancoast syndrome (Pancoast’s syndrome) is characterized by a malignant neoplasm of the superior sulcus of the lung (lung cancer) with destructive lesions of the thoracic inlet and involvement of the brachial plexus and cervical sympathetic nerves (stellate ganglion) which causes Horner syndrome (ptosis, miosis, hemianhidrosis, enophthalmos).

## Objectives

Following trimodality treatment for (SSTs), the 5-year survival rate has significantly improved. The objective of this study was mortality and morbitity and quality of life after the resection of superior sulcus tumours following neoadjuvant chemoradiation.

## Methods

Between Januar 2002 and December 2012 ; 55 consecutive patients were treated with primary radiotherapy followed by surgery or with primary excision and subsequent radiotherapy in the absence of an initial histological diagnosis.

## Results

In total, 55 patients participated in this study (45 men and 10 women). The median age was 60 years (range 40–73), median radiation dose 45 Gy (range 40–60) and median follow-up 50 months (range 4–101). The mortality of patients given the combined treatment was 8.6% (one death due to pulmonary embolism and one death due to myocard ischemia), and the five year survival rate was 20.6% for all patients and 37% for those who underwent complete resection without N2 disease.

## Conclusion

Stage III lung cancer, classified as Pancoast tumour according to strict, consistent criteria, is best treated by primary radiotherapy; combined treatment should be used only for patients with potentially resectable cancer without N2 disease and/or malignant invasion of the first rib.

